# High-frequency hearing loss, occupational noise exposure and hypertension: a cross-sectional study in male workers

**DOI:** 10.1186/1476-069X-10-35

**Published:** 2011-04-25

**Authors:** Ta-Yuan Chang, Chiu-Shong Liu, Kuei-Hung Huang, Ren-Yin Chen, Jim-Shoung Lai, Bo-Ying Bao

**Affiliations:** 1 Department of Occupational Safety and Health, College of Public Health, China Medical University, 91 Hsueh-Shih Road, Taichung 40402, Taiwan; 2 Department of Family Medicine, China Medical University Hospital, 2 Yuh-Der Road, Taichung 40447, Taiwan; 3 Department of Pharmacy, College of Pharmacy, China Medical University, 91 Hsueh-Shih Road, Taichung 40402, Taiwan

## Abstract

**Background:**

The association between occupational noise exposure and hypertension is inconsistent because of an exposure bias caused by outer-ear measurements of noise levels among workers. This study used hearing loss values (HLVs) measured at 4 kHz and 6 kHz in both ears as a biomarker to investigate the chronic effects of noise exposure on hypertension in 790 aircraft-manufacturing workers.

**Methods:**

Participants were divided into a high hearing loss (HL) group (n = 214; average HLVs ≥ 30 decibel [dB] at 4 kHz or 6 kHz bilaterally; 83.1 ± 4.9 A-weighted decibel [dBA]), a median HL group (n = 302; 15 ≤ average HLVs < 30 dB at 4 kHz or 6 kHz bilaterally; 83.1 ± 4.4 dBA) and a low HL group (n = 274; average HLVs < 15 dB at 4 kHz or 6 kHz bilaterally; 82.2 ± 5.1 dBA) based on the results of pure tone audiometry. Multivariate logistic regressions were used to estimate the risk of hypertension between groups.

**Results:**

The prevalence rates of hypertension were significantly higher in the high HL (43.5%; p = 0.021) and median HL (42.1%; p = 0.029) groups than in the low HL group (33.2%). The high HL and median HL workers had 1.48-fold (95% confidence interval [95%CI] = 1.02-2.15; p = 0.040) and 1.46-fold (95%CI = 1.03-2.05; p = 0.031) higher risks of hypertension relative to the low HL workers. Employment duration was significantly and positively correlated with the risk of hypertension among workers with average HLVs ≥ 15 dB at 4 kHz (p < 0.001) and 6 kHz (p < 0.001) bilaterally.

**Conclusions:**

Our findings suggest that high-frequency hearing loss is a good biomarker of occupational noise exposure and that noise-induced hearing loss may be associated with the risk of hypertension.

## Background

Prolonged and repeated exposure to road traffic or aircraft noise is reportedly associated with hypertension [1-10]. Noise, a psychosocial stressor, may cause hypertension by activating the hypothalamic-pituitary-adrenal and sympathetic nervous systems and thus causing elevated levels of adrenaline, noradrenaline and cortisol [11-13]. These three hormones contribute to blood pressure regulation. In occupational settings with noise levels above 85 A-weighted decibels (dBA), the association between occupational exposure and hypertension is inconsistent. Some studies have suggested that occupational noise exposure is associated with a sustained elevation of blood pressure [14-18] or with a higher risk of hypertension [18-20], but other studies have not revealed any significant interaction [21-24]. The difference between these studies may be due to the variable use of personal protective equipment (PPE) among workers in high-noise environments. Thus, outer-ear measurements of noise levels alone may be a source of exposure bias because they do not reflect the true intensity of inner-ear exposure.

Many epidemiological studies have demonstrated that high-frequency hearing loss may be associated with occupational noise exposure. Previous studies have documented that an audiometric notch at 3, 4 or 6 kHz with recovery at 8 kHz is a sign of noise-induced hearing loss [25-28]. In one industrial-based study, exposure to noise levels ≥ 85 dBA for more than 5 years was associated with hearing loss of 28.3 dB at 4 kHz among automotive assembly workers [29].

Few studies, however, have used hearing loss at high frequencies as a biological marker for noise exposure to investigate the risk of hypertension. One field study reported significantly higher means of systolic blood pressure (SBP) and diastolic blood pressure (DBP) in workers with an auditory impairment ≥ 65 dB at 3, 4, or 6 kHz compared with those with normal hearing [30]; however, their results were limited to an arbitrary criterion of hearing loss, and the dose-response relationship between hearing loss and risk of hypertension was unclear. The present study attempted to determine whether high-frequency hearing loss could be used as a biomarker of occupational noise exposure; we also investigated the relationships between occupational noise exposure and hypertension in male workers.

## Methods

### Subjects

In this cross-sectional study, we recruited 948 male workers in an aircraft-manufacturing company with 1,094 employees at the end of 1998 in Central Taiwan. To avoid interference from non-occupational exposure, we used questionnaire answers to exclude 57 workers with previously diagnosed hearing loss, 27 workers who commonly used portable media players or similar technologies and two workers with both exclusion criteria. In addition, 72 office workers were excluded to prevent a possible bias due to the healthy worker effect. Therefore, the study group comprised 790 production-line workers. Production-line workers were exposed to occupational noise due to forging and casting, grinding, hammering, riveting, trimming, peripheral element assembly and engine operation. The present study was reviewed and approved by the Institutional Review Board of China Medical University Hospital before the study commenced. Written informed consent was obtained from each participant.

### Blood pressure measurements and hypertension

All subjects were required to fast overnight before blood sampling and blood pressure measurements. Subjects sat for 10 minutes before blood pressure was measured bilaterally by a trained nurse using an automated sphygmomanometer (Ostar Model P2, Ostar Meditech Corp., Taipei, Taiwan). The mean value of the two blood pressure measurements was used to represent individuals' blood pressure in the present study. Subjects were defined as hypertensive if they reported a previous medical diagnosis of hypertension, if their mean resting SBP was ≥ 140 mm Hg or if their mean resting DBP was ≥ 90 mm Hg. Height, body weight and total cholesterol level were also measured in all subjects. Body mass index (BMI) was calculated as body weight (kg) divided by the square of the height (m^2^).

We also used a self-administered questionnaire to identify potential confounders. These factors included age, educational level, employment duration, tobacco and alcohol use, regular exercise, history of hypertension and use of PPE. Tobacco use was defined as smoking cigarettes on more than three days per week for the last six months; alcohol use was defined similarly. Regular exercise was defined as participating in a sporting activity at least three times per week for six months or more. The use of PPE included the percentage of time that the subjects wore PPE and the type of PPE (i.e., earplugs and earmuffs) used at work.

### Hearing test and noise exposure assessment

We used the pure tone audiometry data from workers' health checkups to assess individual hearing loss. All 790 subjects in the production line underwent pure tone audiometry tests with a pure tone audiometer (Miracle Ear ME-2 Audiometer model 12582, Miracle-Ear Inc., Minneapolis, Minnesota, USA). Both ears were tested using the method of ascending pure tones at frequencies of 0.5, 1, 2, 4 and 6 kHz before descending to 1 and 0.5 kHz. Hearing tests were preceded by a period of at least 14 hours without exposure to occupational noise above 80 decibels (dB). Hearing measurements were performed in a soundproof room that met the American National Standards Institute (ANSI) S 3.1-1991 specifications [31]. The details for hearing loss testing and analyses have been described previously [32].

We measured environmental noise exposure using a sound analyzer (TES-1358, TES Electronic Corp., Taipei, Taiwan) that can report 1-second to 24-hour continuous equivalent sound levels (Leq) in the range of 30-130 dBA as well as time-weighted-average (TWA) noise levels. This equipment was calibrated with a sound-level calibrator (TES-1356, TES Electronic Corp., Taipei, Taiwan) before environmental monitoring. The 15-min TWA Leq was collected by industrial hygienists at 337 locations around the company using short-term environmental sampling. Each subject was assigned a specific value of noise exposure based the Leq measured in that subject's workplace.

To examine the association between chronic noise exposure and the prevalence of hypertension, we used the hearing loss quantified bilaterally at 4 kHz and 6 kHz as a marker for environmental noise exposure to classify the subjects into high hearing loss (HL), median HL and low HL groups. The notch at 4 or 6 kHz in the audiogram is a well-known indicator of noise-induced hearing loss [25]. We chose 15 dB and 30 dB notches at 4 kHz or 6 kHz as the cut-off thresholds between different HL groups because they represented the second and third quartiles in the distribution of bilateral hearing loss values (HLVs) among all participants. The 790 workers were subdivided into a high HL group (n = 214; average HLVs ≥ 30 dB at 4 kHz or 6 kHz bilaterally), a median HL group (n = 302; 15 ≤ average HLVs < 30 dB at 4 kHz or 6 kHz bilaterally) and a low HL group (n = 274; average HLVs < 15 dB at 4 kHz or 6 kHz bilaterally). We also categorized the relationship between hearing loss and employment duration based on the first quartile (five years) and median (10 years) of employment duration among all participants.

### Statistical analysis

We first used the Shapiro-Wilk test to determine the normality of continuous variables, including age, environmental noise level, employment duration, BMI, total cholesterol level and triglyceride level. The statistical p values for these variables were less than 0.001 among all participants, indicating an abnormal distribution. We therefore performed multiple comparisons between the three groups using the Kruskal-Wallis test for continuous variables and the Chi-square test for dichotomous variables. For those groups with significant differences, the Mann-Whitney test and the Chi-square test (or Fisher's exact test) were used to compare the high and median HL groups with the low HL group. In addition, Spearman's rank correlation was used to correlate occupational noise levels with HLVs at 4 kHz and 6 kHz in either ear among all subjects. We used multivariate logistic regressions and calculated odds ratios (ORs) and 95% confidence intervals (CIs) to compare the between-group differences in hypertension while controlling for potential confounding factors. These confounders included age, educational level, BMI (or triglyceride level), tobacco use, alcohol consumption and regular exercise. The SAS standard package for Windows version 9.1 (SAS Institute Incorporation, Cary, North Carolina, USA) was used for statistical analyses. The significance level was set at 0.050 for all tests.

## Results

Table 1 summarizes the demographic characteristics and potential risk factors of the three groups of 790 participants. The mean ages, environmental noise levels and probabilities of PPE use varied significantly among the three groups. The high HL workers were significantly older and had higher environmental noise levels as well as a higher proportion of PPE use at work than the low HL workers. Workers in the median HL group were exposed to significantly higher noise levels and had a higher proportion of PPE use at work compared with those in the low HL group. The more frequent use of PPE by workers in the high and median HL groups might reflect awareness of auditory impairment and a resulting choice to wear PPE. There were no significant differences between these three groups in terms of employment duration, BMI, total cholesterol level, triglyceride level, educational level, tobacco use, alcohol consumption, regular exercise and history of hypertension (p > 0.050).

**Table 1:** Demographic characteristics and risk factors for hypertension in the three study groups

Characteristics	Hearing loss groups			P-value
		
	High	Median	Low	
Subjects (no.)	214	302	274	

Age (years)				

Mean (SD)	41.4 (7.1)^d, e^	38.1 (5.6)	37.4 (6.7)	< 0.001^a^

Environmental noise (dBA)				

Mean (SD)	83.1 (4.9)^d^	83.1 (4.4)^d^	82.2 (5.1)	< 0.001^a^

Employment duration (years)				

Mean (SD)	10.4 (6.0)	9.8 (5.1)	9.1 (5.4)	0.192^a^

Body Mass Index (kg/m^2^)				

Mean (SD)	24.4 (3.2)	23.9 (3.0)	24.0 (3.2)	0.250^a^

Total cholesterol level (mg/dL)				

Mean (SD)	185.7 (33.1)	188.4 (38.5)	183.4 (35.4)	0.372^a^

Triglyceride level (mg/dL)				

Mean (SD)	153.2 (100.6)	147.2 (69.5)	149.9 (84.9)	0.584^a^

Educational level				

< 12 years (%)	143 (66.8)	177 (58.6)	180 (65.7)	0.096^b^

Tobacco use				

Yes (%)	118 (55.1)	154 (51.0)	157 (57.3)	0.304^b^

Alcohol consumption				

Yes (%)	134 (62.6)	180 (59.6)	177 (64.6)	0.460^b^

Regular exercise				

Yes (%)	71 (33.2)	90 (29.8)	90 (32.9)	0.643^b^

Disease history of hypertension				

Yes (%)	5 (2.3)	4 (1.3)	1 (0.4)	0.165^c^

Use of PPE at work				

Never (%)	76 (35.5)^f^	108 (35.8)^f^	150 (54.7)	< 0.001^b^

< 2 hours working time (%)	36 (16.8)	68 (22.5)	50 (18.3)	

2-4 hours working time (%)	35 (16.4)	39 (12.9)	31 (11.3)	

> = 4 hours working time (%)	67 (31.3)	87 (28.8)	43 (15.7)	

dBA = A-weighted decibel; PPE = personal protective equipment; SD = standard deviation.

^a ^Kruskal-Wallis test of the difference between three groups. ^b ^Chi-square test of the difference between three groups. ^c ^Fisher's exact test of the difference between three groups. ^d ^Mann-Whitney test for a significant difference (p < 0.050) compared with the low-hearing-loss group. ^e ^Mann-Whitney test for a significant difference (p < 0.050) compared with the median-hearing-loss group. ^f ^Chi-square test for a significant difference (p < 0.050) compared with the low-hearing-loss group.

Table 2 presents the quantified hearing loss for these three groups and the correlation with noise levels. There were significant differences among the three HL groups in mean HLVs at 4 kHz in left and right ears as well as in those at 6 kHz. The mean bilateral HLVs at 4 kHz and those at 6 kHz were significantly higher in the high and median HL groups than in the low HL group. The high HL workers had significantly higher mean HLVs at 4 kHz and at 6 kHz bilaterally compared with the median HL workers. In addition, workers' exposure to noise levels was significantly correlated with the mean HLVs at 4 kHz and at 6 kHz bilaterally (all p values < 0.001), with the highest correlation at 6 kHz in the left ear (r = 0.172).

**Table 2:** Hearing loss at high frequencies and correlation with noise levels among the three groups

Variable	High HL group	Median HL group	Low HL group	P-value	Spearman's rank correlation	
			
	Median (IRQ)	Median (IRQ)	Median (IRQ)		Coefficient	P-value
Hearing loss (dB) at 4000 Hz						

Left ear	35.0 (30.0)^b, c^	20.0 (15.0)^b^	0 (10.0)	< 0.001^a^	0.169	< 0.001

Right ear	35.0 (35.0)^b, c^	20.0 (10.0)^b^	0 (5.0)	< 0.001^a^	0.149	< 0.001

Mean of both ears	35.0 (27.5)^b, c^	17.5 (10.0)^b^	0 (7.5)	< 0.001^a^	0.159	< 0.001

Hearing loss (dB) at 6000 Hz						

Left ear	40.0 (20.0)^b, c^	20.0 (10.0)^b^	0 (10.0)	< 0.001^a^	0.172	< 0.001

Right ear	45.0 (30.0)^b, c^	20.0 (10.0)^b^	0 (5.0)	< 0.001^a^	0.127	< 0.001

Mean of both ears	42.5 (20.0)^b, c^	17.5 (7.5)^b^	0 (7.5)	< 0.001^a^	0.145	< 0.001

dB = decibel; dBA = A-weighted decibel; HL = hearing loss; IRQ = interquartile range; SD = standard deviation.

^a ^Kruskal-Wallis test of the difference between three groups. ^b ^Mann-Whitney test for a significant difference (p < 0.050) compared with the low-hearing-loss group. ^c ^Mann-Whitney test for a significant difference (p < 0.050) compared with the median-hearing-loss group.

Table 3 shows the prevalence of hypertension and age-adjusted risk by study groups. There was a significant difference between these three groups in the prevalence of hypertension. The high (43.5%; p = 0.021) and median (42.1%; p = 0.029) HL workers had significantly higher prevalence rates of hypertension than the low HL workers (33.2%). The age-adjusted OR for hypertension was 1.50 (p = 0.033) in the high HL group and 1.45 (p = 0.031) in the median HL group compared with the low HL group. Only the mean values of resting DBP in the high and median HL groups were slightly greater than that in the low HL group.

**Table 3:** Prevalence of hypertension and age-adjusted ORs (95% CIs) by study groups

Groups	No.	Resting SBP	Resting DBP	Hypertension No. (%)	OR (95%CI)
				
		Mean ± SD (mm Hg)	Mean ± SD (mm Hg)		
Low-hearing-loss group	274	123.2 ± 11.6	82.1 ± 8.8	91 (33.2)	1.00

Median-hearing-loss group	302	122.7 ± 12.4	82.9 ± 8.8	127 (42.1)^c^	1.45 (1.03-2.04)

High-hearing-loss group	214	122.9 ± 12.7	83.0 ± 9.1	93 (43.5)^c^	1.50 (1.03-2.18)

		p = 0.800^a^	p = 0.444^a^	p = 0.034^b^	

95%CI = 95% confidence interval; DBP = diastolic blood pressure; OR = odds ratio; SBP = systolic blood pressure; SD = standard deviation.

^a ^Kruskal-Wallis test of the difference between three groups. ^b ^Chi-square test of the difference between three groups. ^c ^Chi-square test for a significant difference (p < 0.05) compared with the low-hearing-loss group.

The associations between each group and the risk of hypertension are summarized in Table 4. Because age was significantly correlated with employment duration (r = 0.387, p < 0.001) and BMI had a significant correlation with the triglyceride level (r = 0.214, p < 0.001), only six potential confounders (age, educational level, triglyceride level, tobacco use, alcohol consumption and regular exercise) were included in the final model. The multivariate logistic regression analysis showed that noise-induced hearing loss at high frequency was significantly associated with hypertension even after controlling for potential confounders. Workers with mean HLVs of 37.9 ± 18.7 dB at 4 kHz or 45.9 ± 14.5 dB at 6 kHz bilaterally had a 1.48-fold greater OR for hypertension than those with mean HLVs of 3.6 ± 5.9 dB at 4 kHz or 3.5 ± 4.7 dB at 6 kHz bilaterally; those with mean HLVs of 18.9 ± 8.4 dB at 4 kHz or 18.9 ± 5.4 dB at 6 kHz bilaterally had a 1.46-fold increased OR. We also found that workers with a triglyceride level ≥150 mg/dl were at significantly higher risk for hypertension. In addition, there was a dose-response association between the three HL groups and the risk of hypertension after adjusting for potential confounders (adjusted OR = 1.22, 95%CI = 1.02-1.47; p = 0.033).

**Table 4:** Associations between different hearing loss groups and the prevalence of hypertension

Variables	Univariate OR (95%CI)	P-value	Multivariate OR^a ^(95%CI)	P-value
Median HL group vs. low HL group	1.46 (1.04-2.05)	0.029	1.46 (1.03-2.05)	0.031

High HL group vs. low HL group	1.55 (1.07-2.24)	0.021	1.48 (1.02-2.15)	0.040

Age (years): > = 40 vs. < 40	1.26 (0.94-1.68)	0.123	1.22 (0.91-1.65)	0.191

Educational level (years): > = 12 vs. < 12	1.02 (0.76-1.37)	0.900	1.04 (0.77-1.41)	0.797

Triglyceride level (mg/dl): > = 150 vs. < 150	1.55 (1.11-2.18)	0.011	1.53 (1.09-2.16)	0.015

Tobacco use: yes vs. no	0.98 (0.74-1.31)	0.897	1.13 (0.62-2.04)	0.698

Alcohol consumption: yes vs. no	0.97 (0.72-1.30)	0.846	0.85 (0.45-1.63)	0.631

Regular exercise: yes vs. no	1.08 (0.80-1.47)	0.618	1.17 (0.81-1.67)	0.403

95%CI = 95% confidence interval; HL = hearing loss; OR = odds ratio.

^a^The multivariate logistic regression was used to adjust for age, educational level, triglyceride level (> = 150 vs. < 150 mg/dl), tobacco use, alcohol consumption and regular exercise.

**Figure 1:**
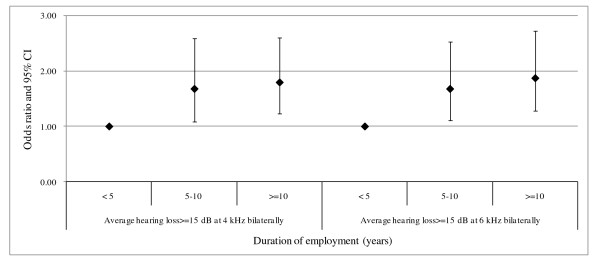
**Odds ratios and 95% confidence intervals for hypertension by duration of employment among workers**. All analyses were adjusted for age, educational level, body mass index, smoking, alcohol consumption and regular exercise.

In order to investigate the threshold above which HLVs could be used as a biomarker and to determine the critical duration of cumulative exposure, the risk of hypertension in workers with 5-10 or greater than 10 years of employment who had average HLVs ≥ 15 dB at 4 kHz or 6 kHz bilaterally were compared with those in the reference group. As shown in Figure 1, 107 workers with average HLVs ≥ 15 dB at 4 kHz bilaterally during 5-10 years of employment (adjusted OR = 1.67, 95%CI = 1.08-2.59; p = 0.021) as well as 155 workers during over 10 years (adjusted OR = 1.79, 95%CI = 1.23-2.61; p = 0.002) had the significantly higher risk of hypertension than the reference group (including 366 workers with average HLVs < 15 dB at 4 kHz bilaterally and 162 workers with HLVs ≥ 15 dB at 4 kHz bilaterally for less than five years of employment) after controlling for age, BMI, tobacco use, alcohol consumption and regular exercise. In addition, 127 workers with average HLVs ≥ 15 dB at 6 kHz bilaterally who had been employed for 5-10 years (adjusted OR = 1.67, 95%CI = 1.11-2.53; p = 0.015) and 154 workers employed for more than 10 years (adjusted OR = 1.87, 95%CI = 1.28-2.73; p = 0.001) had a significantly higher risk of hypertension than the reference group (including 319 workers with average HLVs < 15 dB at 6 kHz bilaterally and 190 workers with HLVs ≥ 15 dB at 6 kHz bilaterally but with less than five years of employment) after controlling for potential confounders. There were significant and positive correlations between employment duration and the risk of hypertension in workers with average HLVs ≥ 15 dB at 4 kHz (adjusted OR = 1.34, 95%CI = 1.14-1.64; p < 0.001) and those with average HLVs ≥ 15 dB at 6 kHz (adjusted OR = 1.40, 95%CI = 1.16-1.68; p < 0.001) bilaterally.

## Discussion

In this study, we used high-frequency hearing loss as a surrogate for occupational noise exposure to assess the association between chronic exposure to noise and the risk of hypertension. We found that the mean HLV measured bilaterally at 4 kHz or 6 kHz was a good indicator of the effects of prolonged occupational noise exposure because it was significantly correlated with outer-ear noise levels and could reveal significant between-group differences in hearing loss despite the confounding effect of PPE use. Our data revealed that high and median HL workers had significantly higher risks of hypertension and slightly greater mean values of resting DBP than low HL workers, although they had a significantly higher proportion of using PPE at work (64.5% and 64.2% vs. 45.3%). In a retrospective cohort study, male workers exposed to a personal noise level ≥ 85 dBA who used both earplugs and earmuffs (96.7%) were found to have a significantly higher mean value of SBP than office workers after a 9-year follow-up [17]. Previous studies reported no significant association between elevated blood pressure and chronic exposure to noise levels above 85 dBA when measured using environmental [21,22] or personal [23] noise monitoring. Such comparisons indicated that assessing occupational noise exposure based on personal or environmental sampling might be subject to exposure biases in occupational settings with more than 85 dBA noise due to variable use of PPE. However, using mean high-frequency HLVs as markers of noise exposure could overcome this bias.

In order to determine the association between noise exposure and hypertension, we also selected 80 dBA and 85 dBA (the second and third quartiles in the distribution of noise levels across workers) as the cut-off values between different noise-exposure groups. We found that only the high-exposure group (n = 348; 86.9 ± 1.6 dBA) had a significantly higher risk of hypertension (adjusted odds ratio = 1.84, 95%CI = 1.29-2.63; p = 0.001) relative to the low-exposure group (n = 301; 77.7 ± 3.4 dBA) after controlling for age, educational level, triglyceride level, tobacco use, alcohol consumption and regular exercise. However, the significant difference between high-exposure and low-exposure groups disappeared (adjusted odds ratio = 1.26, 95%CI = 0.76-2.09; p = 0.381) after adding the single variable representing PPE use to the above multivariate logistic regression. These results showed that the use of PPE confounded analysis of the association between noise exposure and hypertension. Thus, the bilateral means of hearing loss at 4 kHz and 6 kHz were used in the present study instead of outer-ear noise levels.

Like the high-frequency auditory threshold, the distortion product of otoacoustic emissions has also been utilized as an indicator of noise-induced hearing loss in workers [33,34]. Both indicators have been reported to be more sensitive to auditory damage than hearing thresholds at conventional frequencies [33].

Previous studies that investigated the association between noise exposure and hypertension arbitrarily defined a hearing threshold of ≥ 65 dB at 3, 4, or 6 k Hz as a surrogate of noise exposure [30,35]. In this study, we found that the lower mean hearing thresholds of 15 dB and 30 dB at 4 kHz or 6 kHz bilaterally could be used to assess the chronic effects of occupational noise exposure on the risk of hypertension, particularly in noisy environments where workers were required to use PPE.

We also demonstrated a dose-response relationship between the mean hearing threshold at 4 kHz or 6 kHz bilaterally and the risk of hypertension. Previous studies have described the association between hypertension and hearing loss in middle-aged and elderly populations [36,37]. The present study showed that the prevalence rates of hypertension in high and median HL workers differed from that in low HL workers by 10.3% and 8.9%, respectively. In addition, the impact of environmental noise on the prevalence of hypertension was significantly increased across these three groups after adjusting for age, education level, triglyceride level, tobacco use, alcohol consumption and regular exercise. These comparisons indicated that there was a positive association between the hearing loss induced by chronic exposure to occupational noise and the risk of hypertension.

Our data also indicated that employment duration was associated with the risk of hypertension in workers with mean HLVs ≥ 15 dB at 4 kHz or 6 kHz bilaterally. In an industrial-based study, hearing loss at 4 kHz and length of employment in departments with an environmental noise levels > 85 dBA were significantly associated with mean blood pressure and hypertension among black workers with average HLVs of 28.3 ± 16.8 dB and exposure duration of 12.6 ± 6.3 years [29]. We observed a significantly higher risk of hypertension in workers with a bilateral mean HLV of 30.0 ± 16.6 dB at 4 kHz (employment duration of 6.6 ± 1.1 years) and in those with a bilateral mean HLV of 32.1 ± 15.5 dB at 4 kHz (employment duration of 14.8 ± 4.3 years) relative to the reference group. A similar pattern was also found in workers with a bilateral average HLV of 30.9 ± 16.6 dB at 6 kHz and employment duration of 6.7 ± 1.1 years as well as in those with a bilateral average HLV of 33.4 ± 15.7 dB at 6 kHz and employment duration of 14.8 ± 4.2 years.

The application of the bilateral mean HLV at high frequencies in the present study reduced the variability from environmentally sampled noise measurements and from individual use of PPE at work. In addition, this approach minimized information bias caused by misclassification of exposure groups based on the use of the job title or job category [38]. There were similar noise levels and PPE use frequencies among the high HL (83.1 ± 4.9 dBA; 64.5%) and median HL (83.1 ± 4.4 dBA; 64.2%) groups, despite the significant and positive correlation between the three HL groups and the risk of hypertension.

However, a cross-sectional analysis of a temporal problem might restrict the evidence for a causal relationship between noise exposure and hypertension. The study was designed to collect retrospective data over a span of more than 10 years, and the occupational regulation of noise protection in Taiwan has not changed since 1998. Although all of the study subjects had worked in the same environment for more than eight years, the health status of subjects before they were employed was unknown. This limited our ability to elaborate on between-group differences in risk for hypertension due to occupational exposure.

In addition, using hearing loss as a marker of noise exposure instead of direct measurements might be confounded by a genetic tendency to suffer hearing loss and atherosclerosis [39,40]. Atherosclerosis might cause hearing loss and hypertension-induced atherosclerosis might promote hearing loss. Such reverse causality could partially explain the association between hearing loss and hypertension found in this study.

Finally, several potential confounders were not included as covariates in our analyses. Important but uncontrolled risk factors of hypertension include a family history of hypertension, low-density lipoprotein cholesterol and dietary sodium and potassium intake [41,42]. These unmeasured factors might contribute to the sustained difference between these three HL groups.

## Conclusions

Our data showed that high-frequency hearing loss was a good biomarker for occupational noise exposure in aircraft manufacturing workers. A mean hearing threshold exceeding 15 dB at 4 kHz or 6 kHz bilaterally over a 5-year period was associated with an increased risk of hypertension. Future human studies with a follow-up design including participants in different lines of work may find that the bilateral mean hearing threshold at high frequencies is a useful tool for investigating the chronic effects of occupational exposure to noise.

## List of Abbreviations

BMI: Body mass index; dB: Decibel; dBA: A-weighted decibel; DBP: Diastolic blood pressure; HL: Hearing loss; HLV: Hearing loss value; IRQ: Interquartile range; OR: Odds ratio; PPE: Personal protection equipment; SBP: Systolic blood pressure; SD: Standard deviation; 95%CI: 95% confidence interval.

## Competing interests

The authors declare that they have no competing interests.

## Authors' contributions

TYC conceived of the study, developed the study design and drafted the paper. CSL participated in the study together with TYC and helped to draft the manuscript. KHH and RYC performed the statistical analysis and wrote parts of the paper's method section. JSL and BYB participated in design and coordination of the study and supervised the study. All authors of this paper have read and approved the final manuscript.
